# Sepsis Enhances Epithelial Permeability with Stretch in an Actin Dependent Manner

**DOI:** 10.1371/journal.pone.0038748

**Published:** 2012-06-19

**Authors:** Taylor S. Cohen, Brian C. DiPaolo, Gladys Gray Lawrence, Susan S. Margulies

**Affiliations:** Department of Bioengineering, University of Pennsylvania, Philadelphia, Pennsylvania, United States of America; University of Giessen Lung Center, Germany

## Abstract

Ventilation of septic patients often leads to the development of edema and impaired gas exchange. We hypothesized that septic alveolar epithelial monolayers would experience stretch-induced barrier dysfunction at a lower magnitude of stretch than healthy alveolar epithelial monolayers. Alveolar epithelial cells were isolated from rats 24 hours after cecal ligation and double puncture (2CLP) or sham surgery. Following a 5-day culture period, monolayers were cyclically stretched for 0, 10, or 60 minutes to a magnitude of 12% or 25% change in surface area (ΔSA). Barrier function, MAPk and myosin light chain (MLC) phosphorylation, tight junction (TJ) protein expression and actin cytoskeletal organization were examined after stretch. Significant increases in epithelial permeability were observed only in 2CLP monolayers at the 12% ΔSA stretch level, and in both 2CLP and sham monolayers at the 25% ΔSA stretch level. Increased permeability in 2CLP monolayers was not associated with MAPk signaling or alterations in expression of TJ proteins. 2CLP monolayers had fewer actin stress fibers before stretch, a more robust stretch-induced actin redistribution, and reduced phosphorylated MLCK than sham monolayers. Jasplakinolide stabilization of the actin cytoskeleton in 2CLP monolayers prevented significant increases in permeability following 60 minutes of stretch to 12% ΔSA. We concluded that septic alveolar epithelial monolayers are more susceptible to stretch-induced barrier dysfunction than healthy monolayers due to actin reorganization.

## Introduction

Acute lung injury (ALI) is a significant clinical problem with a reported mortality rate between 25% and 40% [1], [2]. It is characterized by acute onset, severe hypoxemia, and pulmonary edema, and can develop following a variety of injuries such as pneumonia, drowning, reperfusion, smoke inhalation, or pulmonary hemorrhage [3]. As respiratory function declines, mechanical ventilation is required to maintain blood gas levels; however, while necessary for survival, ventilation can also injure the lung [4]. Mechanical ventilation has been shown to lead to ALI without the presence of other factors, but is often exacerbated by a predisposing condition [5], such as sepsis [6].

Previous studies have shown a synergistic relationship between ventilation, or stretch, and the expression of inflammatory mediators [7], [8]. Independent studies by Nin *et. al.* and O’Mahony *et. al.* show increases in IL-6 expression following ventilation and either 2CLP in rats or administration of endotoxin (LPS) in mice, above levels observed with either ventilation or sepsis exposure independently. A clinical study on IL-6 conducted by Damas *et. al.* showed a strong correlation between IL-6 expression and patient mortality [9]. Together, these data demonstrate a more injurious systemic effect of the combined insult.

Data identifying a synergistic effect of inflammation and ventilation on the expression of inflammatory cytokines, the development of pulmonary edema, and alveolar epithelial cell mortality have been previously published [10]. Ke-zhong *et. al.* have shown that in combination, intra-tracheal administration of endotoxin (LPS) and ventilation increased pulmonary edema (bronchial alveolar lavage protein concentration and lung wet/dry ratio increased) compared to LPS or ventilation alone [11]. Our lab has demonstrated the combined insult of a cecal ligation and puncture sepsis model and mechanical stretch increases alveolar epithelial permeability in the intact lung, and stretch-induced cell death of alveolar epithelial type II cells in vitro [12], [13]. Taken together, these studies, as well as clinical reports of increased edema fluid in ALI patients, demonstrate that sepsis is associated with a loss of barrier function in the tissue separating the capillary and air spaces.

In the lung, the alveoli are the principal sites of gas exchange. The alveolar wall is composed of opposing cell monolayers, the epithelium and endothelium, which are separated by a thin interstitial space. Of these two cell monolayers, the epithelium is thought to supply the majority of resistance to the motion of ions and proteins into the airways [14]. Previously, we demonstrated that alveolar epithelial cells isolated from the lungs of septic animals form confluent monolayers which exhibit increased permeability, altered tight junction (TJ) protein expression, and increased phosphorylation of mitogen activated protein kinase (MAPk) signaling pathways c-Jun N-terminal kinase (JNK) and extracellular signal-related kinase (ERK) [15]. Furthermore, we have shown that increases in JNK and ERK signaling activation are partially responsible for the permeability increases observed in healthy cells following 60 minutes of stretch at high magnitude (37% change in surface area (ΔSA) or 100% total lung capacity (TLC)) [16]. Therefore, we postulated that sepsis and mechanical stretch would act synergistically to increase epithelial permeability via a MAPk signaling pathway.

In the current study, we tested the hypothesis that the cyclic stretch magnitude required to induce barrier dysfunction would be lower for monolayers formed by cells from septic lungs compared to monolayers formed by healthy epithelial cells. We utilized a previously described culture model of septic epithelia to test the barrier function of septic epithelial monolayers following low (12% ΔSA or 70% TLC) and moderate (25% ΔSA or 90% TLC) magnitudes of cyclic stretch [17], [18]. Furthermore, we hypothesized that MAPk regulation of TJ protein expression and disruption of the actin cytoskeletal organization are mechanisms of barrier dysfunction, and evaluated these mechanisms with inhibitors to MAPk activation and actin depolymerization.

## Methods

### Ethics Statement

All animal use was done in accordance with, and with the approval of, the IACUC in the Office of Regulatory Affairs of the University of Pennsylvania.

### Cecal Ligation and Double Puncture

Male Sprague-Dawley rats (Charles River, Boston, MA) weighing 240–260 grams underwent cecal ligation and double puncture (2CLP) or control (sham) surgery as previously described [13]. Mortality rate 24 hours following the 2CLP procedure was approximately 10%.

### Alveolar Epithelial Type II Cell Isolation

Twenty-four hours following the 2CLP or sham procedures, animals were sacrificed and alveolar epithelial type II cells isolated and cultured for 5 days as previously described [17]. Following 5 days in culture, the cells expressed markers of a type I alveolar epithelial phenotype (RT1-40 and plasminogen activator inhibitor-1 (PAI-1)) and formed confluent monolayers with intact tight junctions [15].

### Cell Monolayer Stretching Protocol

Monolayers (4–5 days in culture) were designated as stretched or unstretched groups in one of four conditions: DMEM, DMEM and a MAPk inhibitor, DMEM and vehicle control (DMSO), or DMEM and an inhibitor to actin depolymerization (Jasplakinolide, 1 µM) [19]. Monolayers were (1) stretched for 0 or 60 minutes to 12% or 25% ΔSA and used to test monolayer permeability; (2) stretched for 0, 10, or 60 minutes to 12% or 25% ΔSA, and then lysed for assessment of MAPk and myosin light chain kinase (MLCK) phosphorylation and TJ protein expression level; or (3) stretched for 0 or 60 minutes to 12% or 25% ΔSA and used to visualize actin organization. The stretch device has been previously described and was operated in an incubator maintained at 37°C [20].

### Monolayer Permeability Assessment

Paracellular permeability was assessed in 2CLP and sham monolayers by monitoring binding of the fluorescent tracer BODIPY-ouabain (approximate radius 20 Å) to basal Na^+^/K^+^-ATPase pumps which are inaccessible to the apically applied tracer when the monolayer is intact [21]. The percent area stained on each image was compared for 2CLP and sham monolayers with and without stretch using an ANOVA and post-hoc Student’s t-test for individual comparisons (N≥3 isolations). Significance was defined as p<0.05 with a Bonferonni correction (β = 4).

### Triton Solubility of Cytoskeletal Associated Proteins

Unstretched 2CLP and sham monolayers were lysed in incubation buffer (phosphate buffered saline, 140 mM NaCL, 5.3 mM KCL, 0.35 mM Na_2_PO_4_, 0.35 mM KH_2_PO_4_, 0.8 mM MgCl_2_, 2.7 mM HEPES) with protease inhibitors at 4 degrees C, homogenized, and the nucleus and large debris removed by centrifugation (10 min, 10 Kg). The supernatant was ultracentrifuged (1 hour 100 Kg) to separate the cytoskeletal fraction (pellet) from the cytosol. The pellet was re-suspended in incubation buffer containing 0.1% triton X-100 for 30 min at 4 degrees C. Following this incubation, the sample was re-centrifuged, the supernatant was collected (soluble fraction), and the pellet was re-suspended in incubation buffer and stored (insoluble fraction). Tight junction protein levels were analyzed by Western blot as described below and the ratio of soluble to insoluble protein was determined by densitometry.

### Western Analysis of MAPk and MLCK Phosphorylation and TJ Proteins

Following stretch, whole cell lysate was collected from 2CLP and sham monolayers (N≥3 isolations for each protein at each stretch condition and treatment) and MAPk phosphorylation and expression of TJ proteins was analyzed via western blot as previously described [15]. MLCK phosphorylation was assayed using antibody to MLCK pS1760 (Life Technologies, Grand Island, NY). Band intensity of actin was used to normalize between lanes. Significant differences across conditions were determined through individual ANOVAs for each signaling pathway and junctional protein, with post-hoc Student’s t-tests with a Bonferonni correction (β = 4) for multiple comparisons used to test between 2CLP and sham populations at each condition.

### Immunofluorescence

Monolayers (N = 2 monolayers per group from each of 2 isolations per group) were fixed and actin fibers were stained with rhodamine-phalloidin and imaged as previously described [22]. Na^+^/K^+^-ATPase pump distribution on the basal surface was assessed using antibody to mouse Na^+^/K^+^-ATPase alpha-1 (Millipore, Billerica, MA).

## Results

### Epithelial Permeability Following Stretch

**Figure 1:**
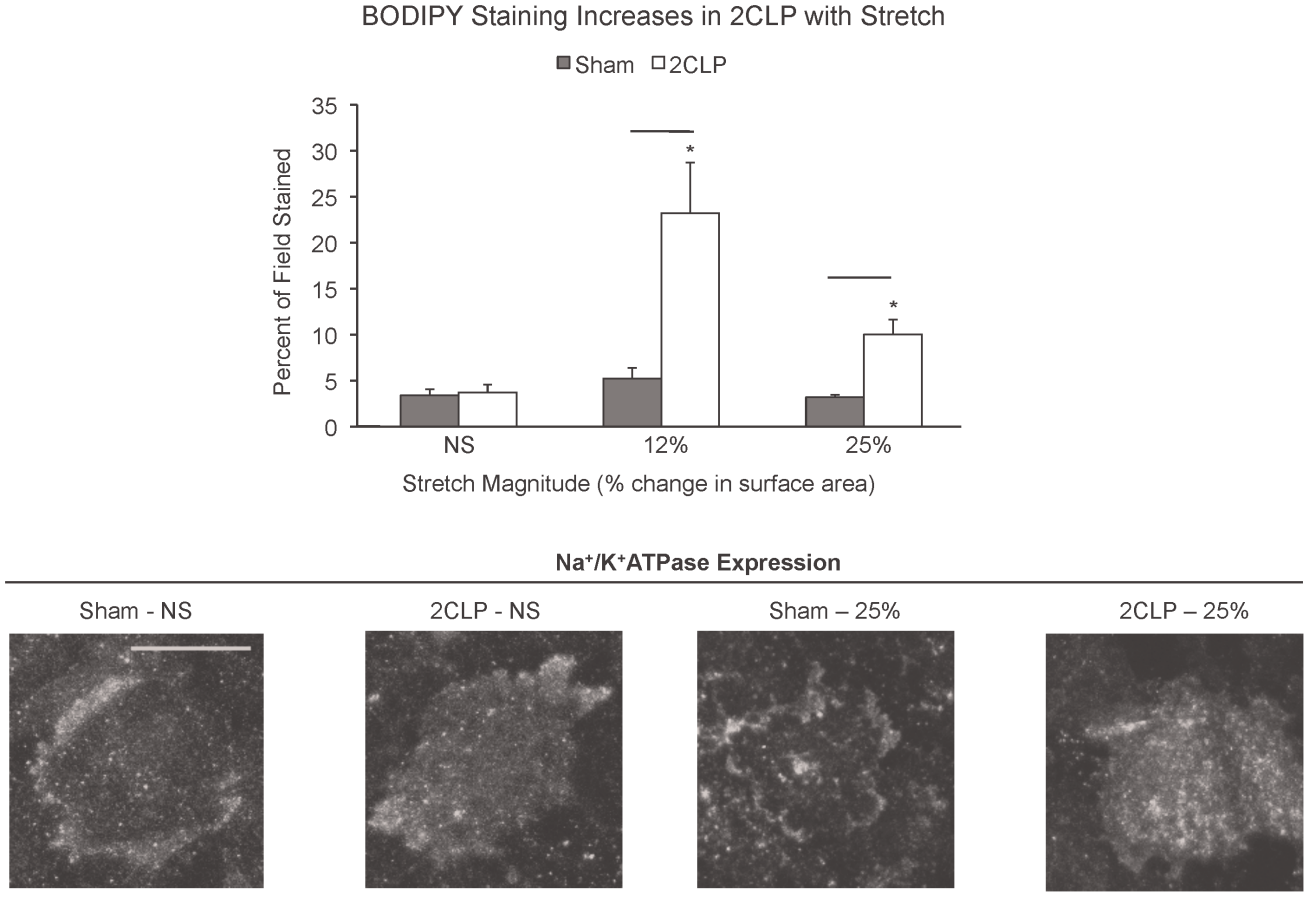
BODIPY staining increases with stretch magnitude. The area (μ ± SE) stained by BODIPY-conjugated ouabain was determined in sham and 2CLP monolayers following 1 hour of cyclic stretch (0.25 Hz) to 0% (NS), 12%, or 25% ΔSA (N≥3 isolations). Area stained in sham monolayers did not increase above unstretched levels, while stained areas in 2CLP monolayers significantly increased above unstretched control levels following stretch to both magnitudes. Confocal micrographs of Na+/K+-ATPase pumps on the basal membrane of unstretched and stretched (1 hour, 25% ΔSA) 4-day old sham and 2CLP monolayers demonstrated that increases in BODIPY staining was not due to differences in receptor expression (Scale bar in Sham – NS = 100 µm). Significant differences between stretch groups were determined by Tukey tests. Significance defined as p<0.01, bar indicates significant difference between sham and 2CLP at same stretch magnitude, *significantly different than 2CLP unstretched. (mean ± SE).

Cultured 2CLP and sham monolayers were stretched for 60 minutes to magnitudes of 0%, 12%, and 25% ΔSA, after which epithelial permeability was immediately analyzed (Figure 1). Without stretch, we observed no significant differences between 2CLP and sham permeability to BODIPY-ouabain (20 Å tracer). Following 60 minutes of stretch to 12% or 25% ΔSA, sham monolayer permeability was unchanged compared to unstretched. Permeability of 2CLP monolayers was significantly greater than unstretched sham and stretched sham controls at both stretch magnitudes. These data show that the stretch magnitude required to alter permeability is lower in 2CLP monolayers compared to sham. Importantly, the percentage of the field stained with BODIPY in sham monolayers is similar to the values previously reported in unstretched and stretched healthy monolayers, while values from stretched 2CLP monolayers are more comparable to a healthy monolayer stretched to 37% ΔSA [21]. Expression of Na^+^/K^+^-ATPase pumps was not different between sham and 2CLP at unstretched or stretched (25% ΔSA for 60 min) conditions indicating that an increase in BODIPY binding sites was not responsible for increased staining (Figure 1).

### Phosphorylation of MAPk Proteins

**Figure 2:**
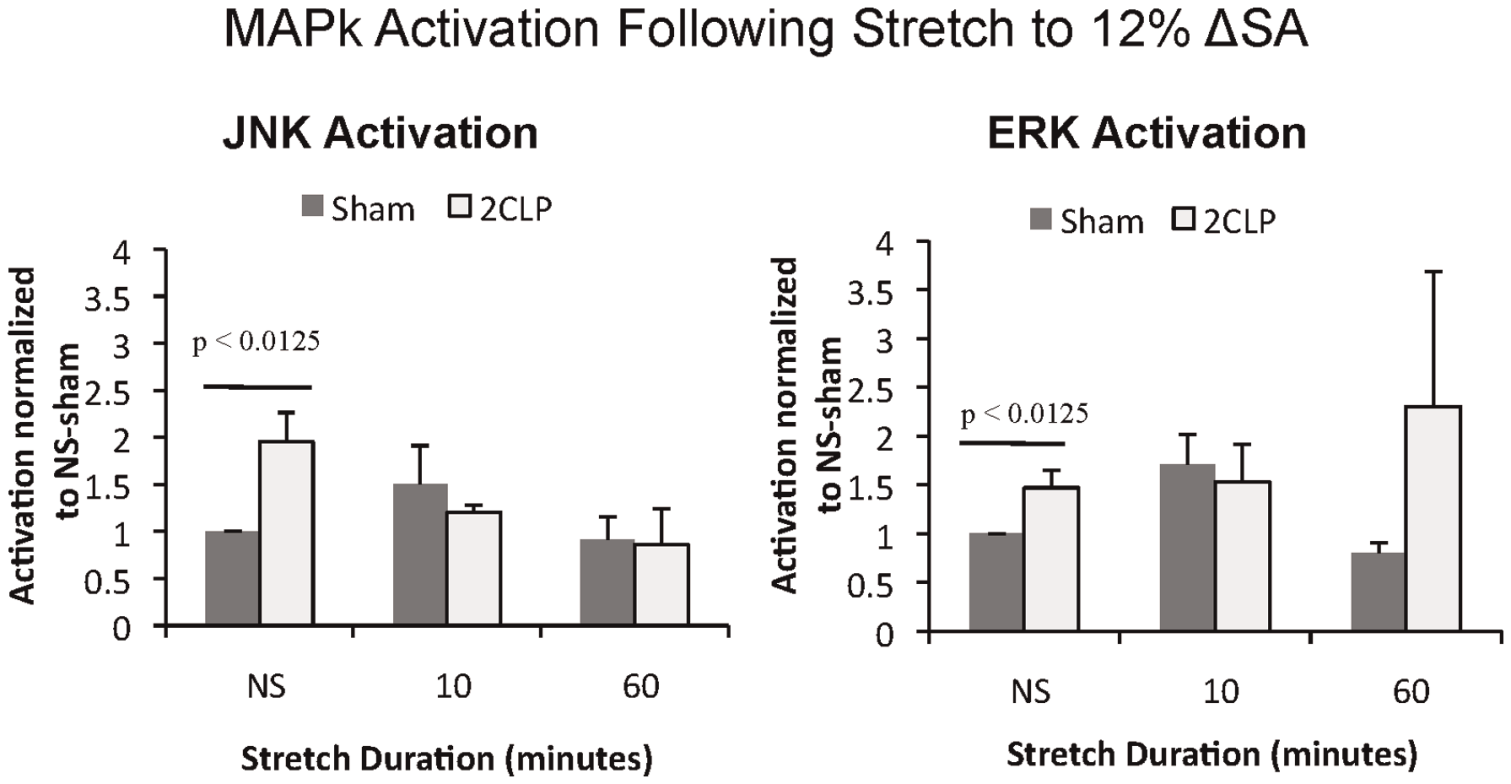
Phosphorylation of MAPk signaling pathways (ERK and JNK) following cyclic stretch for 1 hour to 12% ΔSA in sham and 2CLP cell monolayers. No increases in MAPk phosphorylation were observed in either cell population when stretched to 12% ΔSA for 10 or 60 minutes. (mean ± SE, N≥6 isolations).

Phosphorylation of JNK, ERK, and p38 MAPk was analyzed after 0, 10, and 60 minutes of stretch to 12% and 25% ΔSA. As reported previously, in unstretched monolayers JNK and ERK phosphorylation was significantly increased in 2CLP monolayers compared to sham (bar, Figure 2) [15]. Stretch of 2CLP and sham monolayers to 12% ΔSA did not produce significant increases in JNK, ERK, or p38 phosphorylation (Figure 2). We concluded that activation of MAPk signaling was not responsible for the increased permeability of 2CLP monolayers following 60 minutes of stretch to 12% ΔSA.

A larger stretch magnitude of 25% ΔSA resulted in significant increases in the phosphorylation of JNK and ERK in sham monolayers compared to unstretched sham controls (Figure S1), and significant increases in the phosphorylation of ERK in 2CLP monolayers compared to unstretched sham and 2CLP controls. Phosphorylation of p38 was not observed in 2CLP or sham monolayers at either stretch magnitude (data not shown).

### Tight Junction Protein Expression

**Figure 3:**
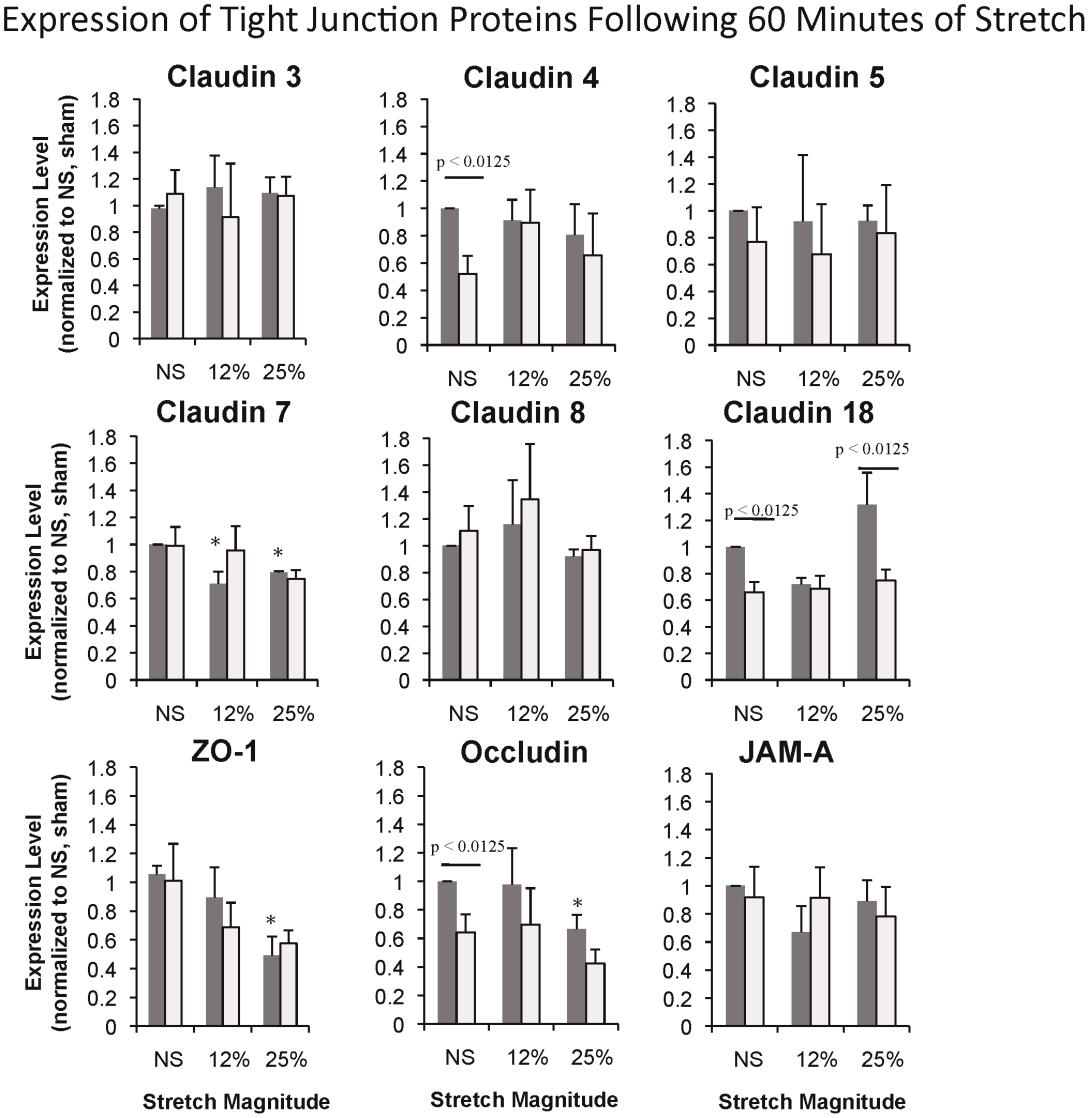
Expression of tight junction proteins following 60 minutes of stretch. Tight junction protein expression in sham (▪) and 2CLP (□) with and without stretch. *significantly different from sham, bar indicates significantly different between sham and 2CLP, p<0.05 (mean ± SE, N≥3 isolations).

The transmembrane proteins of the tight junction, including claudins 3, 4, 5, 7, 8, and 18, occludin, and JAM-A, are ultimately responsible for regulating paracellular permeability, and therefore we analyzed their expression levels following stretch. As reported previously [15], we again observed significantly reduced expression of claudin 4, claudin 18, and occludin levels in whole cell lysates from unstretched 2CLP monolayers compared to sham (NS bars, Figure 3). Stretch to a magnitude of 12% ΔSA did not significantly alter the expression levels of any of the TJ proteins probed in 2CLP monolayers (Figure 3). Low magnitude stretch significantly reduced only the expression of claudin 7 in sham monolayers compared to unstretched. Therefore we concluded that loss of tight junction expression in 2CLP monolayers was not responsible for the observed permeability increases at low stretch magnitudes. Interestingly, we did observe decreases in claudin 7 and ZO-1 expression in sham monolayers following high stretch magnitudes (Figure 3). Inhibition of ERK phosphorylation during high magnitude stretch with U0126 had no effect on permeability or tight junction expression in 2CLP monolayers (Figures S2 and S3). In contrast, ERK inhibition in sham monolayers prevented stretch-induced changes in permeability and in Z01 protein expression.

### Actin Staining

**Figure 4:**
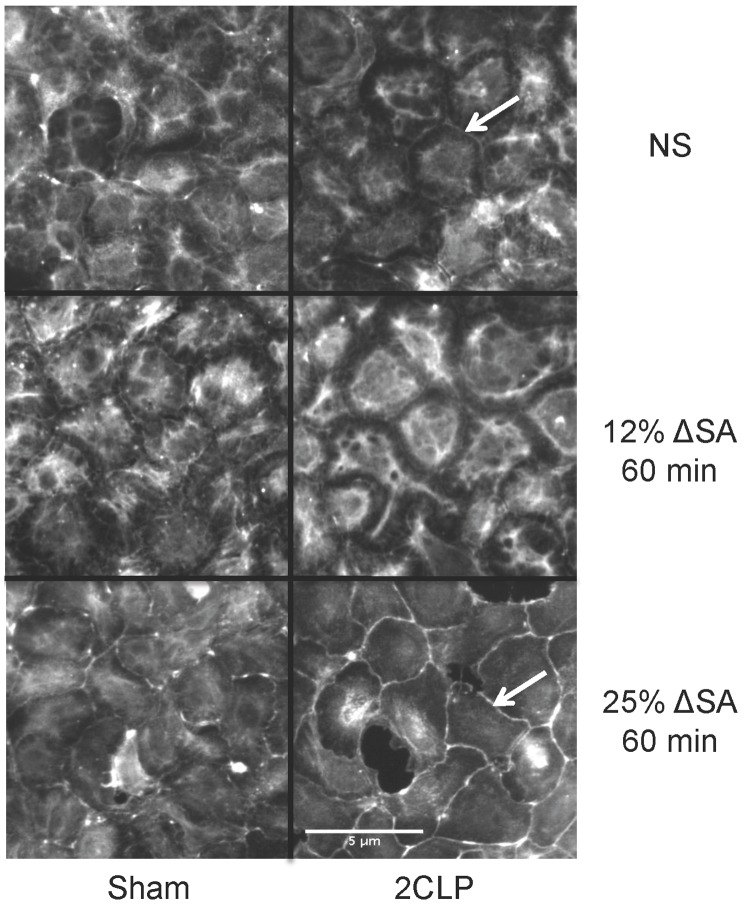
Phalloidin staining of actin in sham and 2CLP monolayers in unstretched, stretched cyclically for 60 minutes to 12% ΔSA, and stretched cyclically for 60 minutes to 25% ΔSA wells. Similar staining patterns were observed in sham unstretched monolayers and sham monolayers stretched to 12% ΔSA. Intensity of the actin stain in unstretched 2CLP monolayers was reduced compared to unstretched sham, and a thin cortical actin ring is apparent (arrow). In 2CLP monolayers stretched to 12% ΔSA, stress fibers form a circular band throughout the cytoplasm of the cell, and become more prominent compared to unstretched monolayers. In cells stretched to 25% ΔSA, cortical rings form (arrow), which are also more prominent in 2CLP monolayers compared to sham. Scale bar 100 µm. Representative of images from 2 isolations with 2 wells per isolation.

Degradation of the actin cytoskeleton has been shown to negatively affect barrier function through alterations in the tight junction-actin associations [23]. Therefore, we labeled actin with phalloidin to visualize its localization within 2CLP and sham monolayers (Figure 4). In unstretched 2CLP and sham monolayers, the actin networks were similar, with diffuse staining through the center of the cell, which extended to the cell periphery in sham monolayers. However, in unstretched 2CLP monolayers, staining at the cell-cell junction was diminished, and only a thin band of actin was observed. Following 12% ΔSA stretch for 60 minutes, actin in sham monolayers was indistinguishable from that in unstretched sham. In 2CLP monolayers, the staining in the cytoplasm increased in intensity, and the junctional staining diminished compared to unstretched 2CLP. Interestingly, circumferential stress fibers became prominent in the cytoplasm of 2CLP cells. Following 25% ΔSA stretch for 60 minutes, cortical actin rings began to form near the cell periphery in sham monolayers; however, staining at the junction was discontinuous. In 2CLP monolayers, bright cortical rings were formed at the cell-cell junctions, while staining intensity decreases in all other areas of the cell. These differences in actin staining in 2CLP and sham monolayers following stretch indicate alterations in cytoskeletal arrangement, even at 12% ΔSA.

### Myosin Light Chain Kinase Phosphorylation

**Figure 5:**
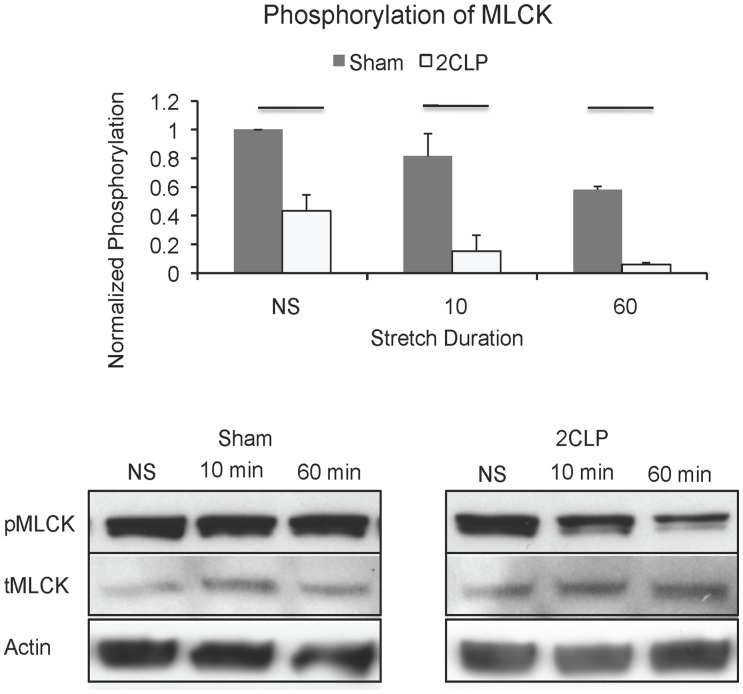
Phosphorylation of MLCK following low-magnitude stretch (12% ΔSA) of sham and 2CLP monolayers. Phosphorylation of MLCK was consistently reduced in 2CLP but not sham monolayers following 60 minutes of cyclic stretch (p<0.05, bar). Total levels of protein (tMLCK) remained unchanged in the cell monolayers. Actin is shown as a loading control. (N = 3 isolations, representative western blots shown).

We hypothesized that alterations to the actin cytoskeleton are responsible for the stretch-induced increases in 2CLP monolayer permeability at low stretch (12% ΔSA). Therefore we analyzed the phosphorylation of myosin light chain kinase (MLCK), a signaling molecule that directly regulates actin cytoskeletal contractility through activation of myosin motors, and a pathway we have demonstrated to be altered during stretch [24]. We found reduced levels of MLCK phosphorylation in unstretched 2CLP monolayers compared to sham monolayers (Figure 5). Stretch to 12% ΔSA reduced MLCK phosphorylation levels compared to unstretched controls in both sham and 2CLP monolayers; however, phosphorylation was consistently lower in 2CLP compared to sham.

### Tight Junction Protein Solubility

**Figure 6:**
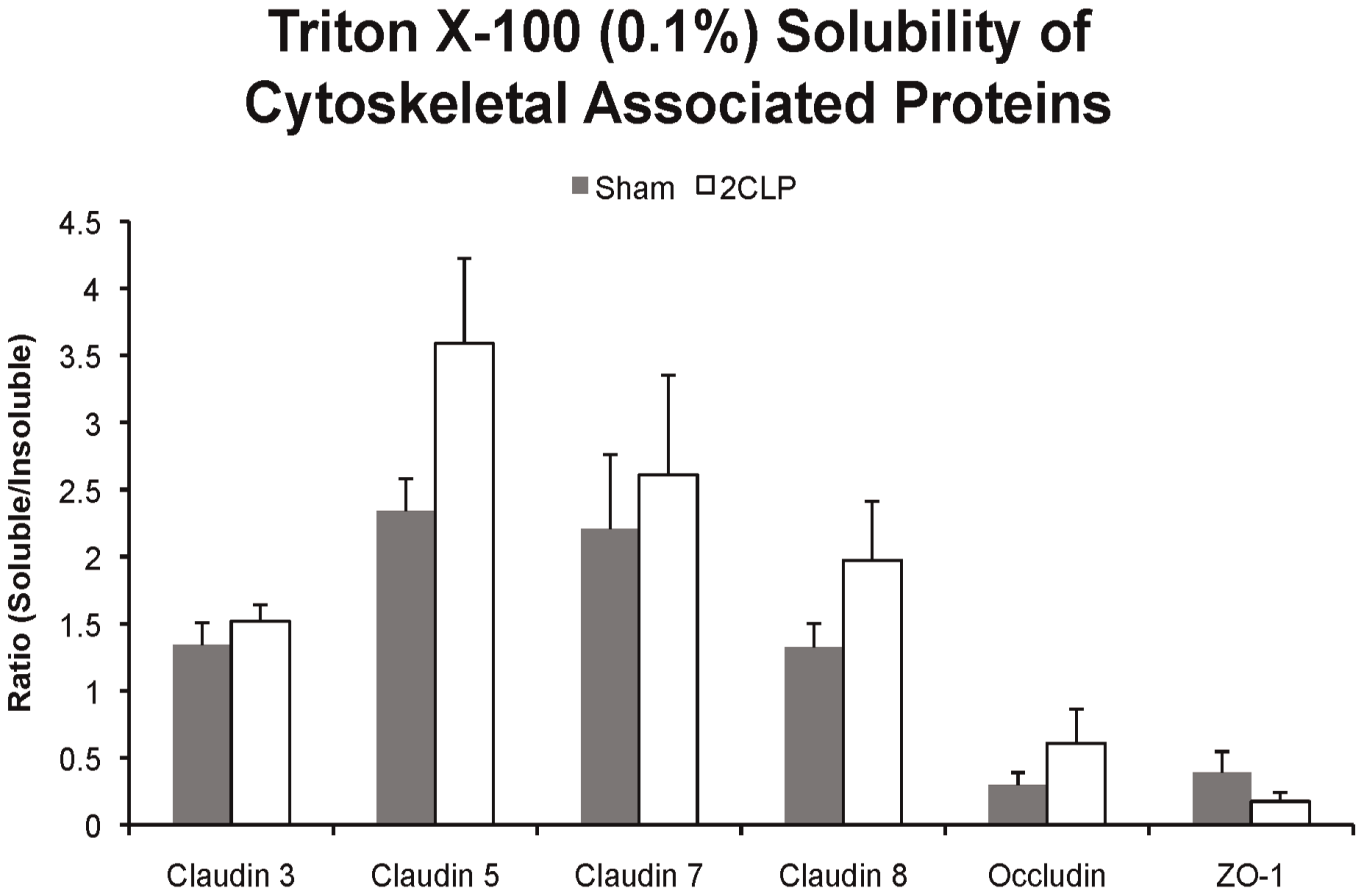
Solubility of cytoskeleton-associated tight junction proteins in unstretched monolayers. The ratio of the amount of soluble to insoluble junctional protein that precipitated with the cytoskeleton of sham (▪) and 2CLP (□) monolayers was higher in the 2CLP population, but did not reach significance. (mean ± SE, N = 4 isolations).

Because our data suggested that the actin cytoskeleton was altered in 2CLP monolayers, we tested the hypothesis that TJ proteins were not anchored to the cytoskeleton in 2CLP monolayers as well as they were in sham monolayers by analyzing the ratio of triton soluble to triton insoluble cytoskeletal associated protein. We found that in unstretched 2CLP monolayers, a greater fraction of the transmembrane proteins dissociated from the cytoskeleton (Figure 6). This result could be indicative of a less organized junctional complex and further studies will be required to determine the nature of the actin-tight junction protein bond in septic epithelial monolayers.

### Stabilization of the Cytoskeleton

**Figure 7:**
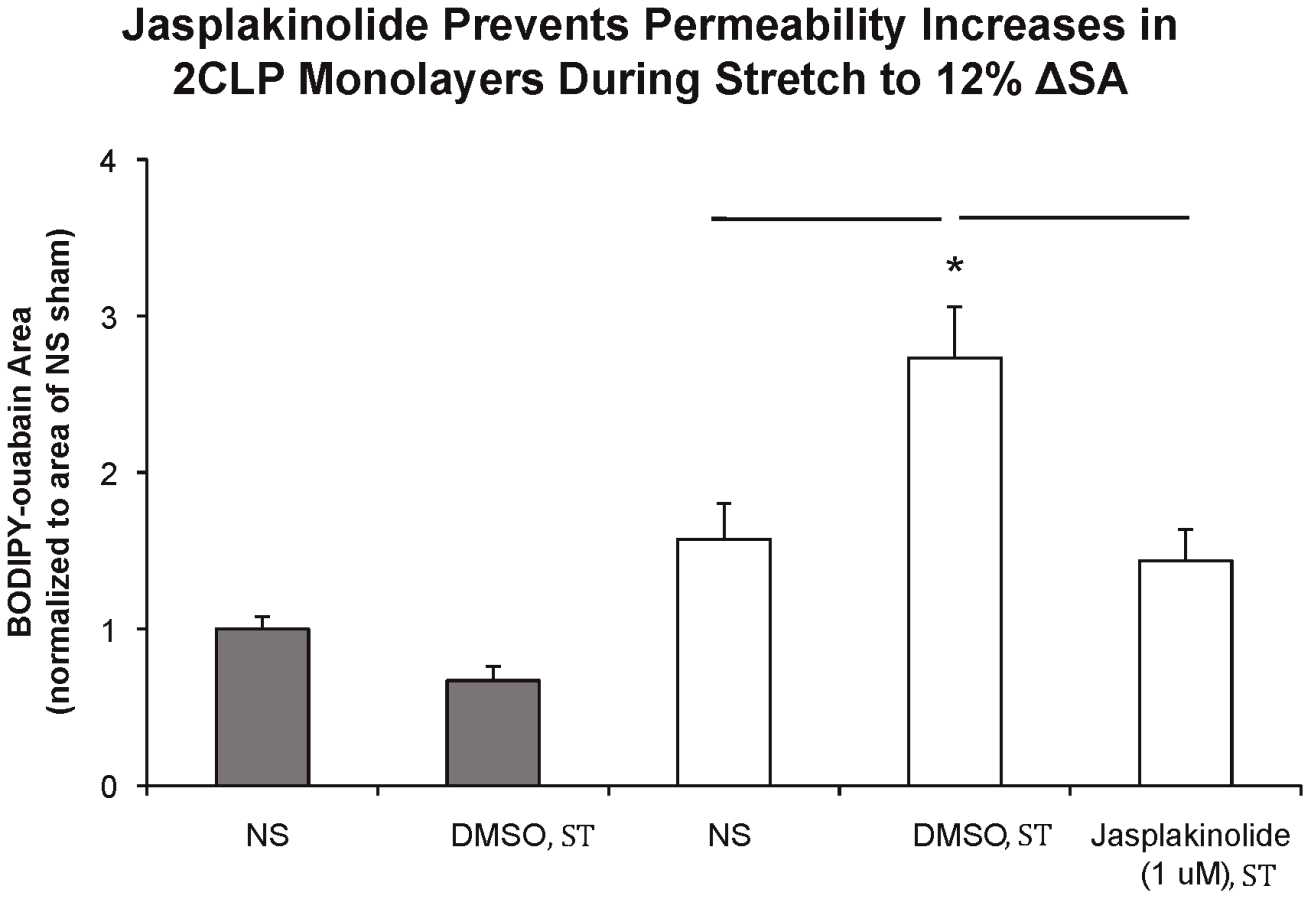
Sham and 2CLP monolayers were cyclically stretched to a magnitude of 12% ΔSA for 60 minutes following a 1-hr incubation with either Jasplakinolide (1 µM, 2CLP) or DMSO (Sham and 2CLP), and permeability to BODIPY-ouabain was analyzed. Stretch resulted in significant permeability increases in DMSO-treated 2CLP monolayers only, compared to unstretched sham and 2CLP monolayers. Treatment with Jasplakinolide prevented all permeability increases. Significance defined as p<0.05 with multiple comparison correction (β = 4), *significantly different than stretched sham (mean ± SE, N≥3 isolations).

To test the hypothesis that actin destabilization in 2CLP monolayers was responsible for the stretch-induced permeability increases at low stretch magnitudes, we used jasplakinolide to prevent actin depolymerization. In previous studies, jasplakinolide has been shown to prevent stretch-induced barrier dysfunction in healthy cell monolayers stretched to 37% ΔSA for 1 hour [12], [19]. For 60 minutes prior to stretch, 2CLP monolayers were treated with jasplakinolide (1 µM) to stabilize the actin cytoskeleton, then cyclically stretched to 12% ΔSA for an additional 60 minutes, after which monolayer permeability was assessed (Figure 7). As shown previously (Figure 1), monolayer permeability was significantly increased in 2CLP and not in sham monolayers treated with DMSO control. Jasplakinolide treatment of 2CLP monolayers preserved barrier properties such that permeability in stretched 2CLP wells treated with jasplakinolide was not significantly increased above unstretched sham or 2CLP levels, and was significantly lower than permeability levels in 2CLP monolayers treated with DMSO. These data show that alterations in the actin cytoskeleton in 2CLP monolayers may be associated with the stretch-induced permeability observed in these cells following even low magnitudes of cyclic stretch.

## Discussion

Ventilation of septic patients is necessary to maintain proper blood gas levels; however, the combination of sepsis and ventilation often leads to the development of acute lung injury (ALI) [2]. A symptom of ALI is the breakdown in epithelial barrier function, which results in edema formation in the airspaces of the lung [25]. We previously demonstrated that stretch to a high magnitude (100% TLC) increases monolayer permeability and activates JNK, ERK, and p38 MAPk in healthy cells, and that inhibition of JNK and ERK reduces the stretch-induced permeability increases [16]. Furthermore, we have developed a model of an epithelium formed by cells from a septic environment and demonstrated that even without stretch these monolayers are less restrictive to paracellular ion flux and have increased phosphorylation of JNK and ERK MAPk [15]. Inhibition of ERK, not JNK, increases monolayer resistance to ion flux in these unstretched septic monolayers. We hypothesized that since sepsis and mechanical ventilation independently activate a common signaling pathway (MAPk) which affects epithelial permeability, the combined insult would then result in an amplified signal leading to barrier dysfunction, even at sub-injurious magnitude.

We showed that in a culture model of the alveolar epithelium, the threshold stretch magnitude above which permeability to a 20 Å tracer increases is lower in monolayers formed from cells isolated from septic animals compared to sham animals. These data confirm previous results from the ex vivo whole lung in which flux of a fluorescent tracer (FITC labeled albumin, 60 Å) across the endothelium and epithelium increases in lungs from 2CLP animals, not sham, ventilated with a tidal volume of 20 ml/kg body weight (∼25% ΔSA) for 30 minutes [12], [18]. Therefore intact lung and isolated cell preparations demonstrated stretch-induced barrier dysfunction in the epithelium of septic animals at levels where healthy epithelia are unaffected.

We previously observed both sepsis and mechanical deformation activate common MAPk signaling pathways. Studies in the intact animal have identified epithelial MAPk signaling as being activated following both large tidal volume ventilation (ERK, JNK) in rats and 2CLP (ERK, JNK, p38) in mice [26], [27]. Additional studies have shown activation of MAPk signaling in vitro following bacterial stimulation, administration of endotoxin, and cyclic stretch [28]–[32]. Based on this body of literature, we hypothesized that the MAPk pathways would modulate the synergistic response to simultaneous insults. When we probed for activation of JNK, ERK, and p38 MAPk in 2CLP and sham monolayers following stretch to 12% ΔSA, we observed no significant phosphorylation of any MAPk above unstretched levels. We conclude that the dual insult of stretch and sepsis is not associated with enhanced MAPk signaling activation in our model, which is counter to our hypothesis of additive activation. Interestingly we did observe MAPk activation following stretch to 25% ΔSA, however inhibition of MAPk signaling in 2CLP monolayers did not prevent stretch-induced permeability increases, suggesting that an alternative mechanism is responsible for the increase in permeability.

The tight junction is an apically located protein complex that controls paracellular permeability of epithelial monolayers, and we hypothesized that the expression levels of TJ proteins, which determine how restrictive the tight junctions are to paracellular fluid and ion motion, would change with injurious levels of stretch. However, we observed no significant alterations in protein expression in 2CLP monolayers following stretch to 12% ΔSA, despite increases in monolayer permeability. Therefore, we conclude that stretch-induced (60 minutes) permeability changes observed in 2CLP monolayers are not due to loss of TJ proteins.

The actin cytoskeleton is integral to the formation and maintenance of the tight junction barrier, and depolymerization, or reorganization, of the actin cytoskeleton has been linked to disruption of junctional integrity in epithelial cells [23], [33]–[39]. Therefore we hypothesized that regulation of the actin cytoskeleton is compromised in 2CLP monolayers, and that cytoskeletal alterations in 2CLP monolayers result in the permeability increases observed following stretch to 12% ΔSA. Visualization of actin stress fibers demonstrated organizational differences between 2CLP and sham cytoskeletons with and without stretch. We further demonstrate that with low magnitude stretch in 2CLP monolayers, the phosphorylation decrease of MLCK may indicate an alteration in actin contractile force generation during deformation. MLCK is a Ca^2+^ dependent signaling molecule that is activated by calmodulin [40]. Phosphorylation of MLCK on serine-1760 inhibits binding of calmodulin to MLCK, preventing its activation [41], [42]. Therefore, in 2CLP monolayers, MLCK is potentially more readily activated in response to changes in intracellular Ca^2+^ due to reduced regulatory signaling. Studies by Turner and colleagues have demonstrated that constitutive MLCK activation increases permeability and destabilizes the junction of non-pulmonary cell lines [43], [44]. Consequently it is not unreasonable to speculate that the lower phosphorylation levels of the regulatory site of MLCK in 2CLP monolayers could contribute to altered barrier function following stretch.

Furthermore, we observe that the cytoskeletal-tight junction complex is more detergent-soluble in unstretched 2CLP monolayers, as well as a significant decrease in occludin expression in 2CLP monolayers compared to sham without stretch, as reported previously [15]. Occludin has been shown to regulate the function of tight junctions through its binding with other TJ proteins [45], [46]. Occludin has been shown to directly interact with claudins, ZO-1, and actin at its C-terminus, and we speculate that with sepsis, the reduction in occludin expression, even without stretch, leads to a more disorganized and leaky tight junction [47].

Jasplakinolide prevents the depolymerization of actin fibers, and has been shown to reduce stretch-induced permeability increases in healthy epithelial monolayers cyclically stretched to 37% ΔSA for 1 hour [12], [48]. Furthermore, treatment with jasplakinolide has recently been shown by our lab to prevent formation of cortical actin rings in healthy primary epithelial cells cyclically stretched to 25% ΔSA for 10 minutes [22]. Pretreatment with jasplakinolide prevented significant increases in 2CLP monolayer permeability following low-level mechanical stretch. Future studies will focus on elucidating the relationship between acto-myosin tension and anchoring of the tight junction complex in septic and healthy epithelial monolayers.

In conclusion, we have shown that cultured epithelial monolayers composed of cells isolated from septic animals have a lower threshold for cyclic stretch-induced barrier dysfunction than one composed of cells from control animals The lower threshold is not due to enhanced activation of MAPk signaling pathways as hypothesized, but rather due to alteration in the actin cytoskeleton organization in septic monolayers. Furthermore, our data suggest that the proteins in the tight junction complex are more weakly associated with the cytoskeleton in monolayers composed of cells from septic animals. These data highlight a need for understanding how sepsis alters the tight junction, the actin cytoskeleton, and the molecules that regulate barrier properties.

## Supporting Information

Figure S1 Stretch to 25% ΔSA induced significant phosphorylation of JNK in sham cells at 60 minutes compared to unstretched (NS) (*, p<0.05), but did not induce JNK phosphorylation at either time point. Stretch to 25% ΔSA for 10 minutes induced significant ERK phosphorylation in both sham and 2CLP monolayers compared to unstretched sham only (*, p<0.05), or to both unstretched sham and unstretched 2CLP (*,#, p<0.05). At 60 minutes of stretch, ERK phosphorylation had subsided in 2CLP monolayers, and was no longer significant from any other time point, while ERK phosphorylation in sham monolayers was still significantly elevated above unstretched sham. (mean ± SE, N≥6 isolations).
(TIFF)

Figure S2 **Inhibition of MAPk signaling (JNK-SP600125 20 µM, ERK-U0126 10 µM) did not prevent stretch-induced permeability increases in 2CLP monolayers when stretched (ST) to 25% ΔSA.** ERK inhibition prevented significant permeability increases in sham monolayers only, when stretched to 25% ΔSA. JNK inhibition did not prevent significant permeability increases in sham and 2CLP monolayers stretched to 25% ΔSA. All stretched for 60 minutes. *significantly greater than sham unstretched (NS), #significantly greater than both sham and 2CLP NS, p<0.05 (mean ± SE, N≥2 isolations).
(TIFF)

Figure S3 **Inhibition of ERK activation in sham monolayers during stretch to 25% ΔSA for 60 minutes significantly reduces expression of claudin 18 compared to levels during stretch with DMSO treatment (θ).** The stretch-induced reduction in sham treated with DMSO ZO-1 is reversed with ERK inhibition; however, this difference was not statistically significant. In 2CLP monolayers, inhibition of JNK activation during stretch significantly reduced occludin levels compared to unstretched (NS) 2CLP (#). *significantly different from DMSO NS, bar indicates significantly different from sham, p<0.05 (mean ± SE, N≥3 isolations).
(TIFF)
