# Supplementary figures and images for: Sepsis Enhances Epithelial Permeability with Stretch in an Actin Dependent Manner
Source: PLoS One. 2012 Jun 19;7(6):e38748. doi: 10.1371/journal.pone.0038748 (PMC3378620; doi:10.1371/journal.pone.0038748)

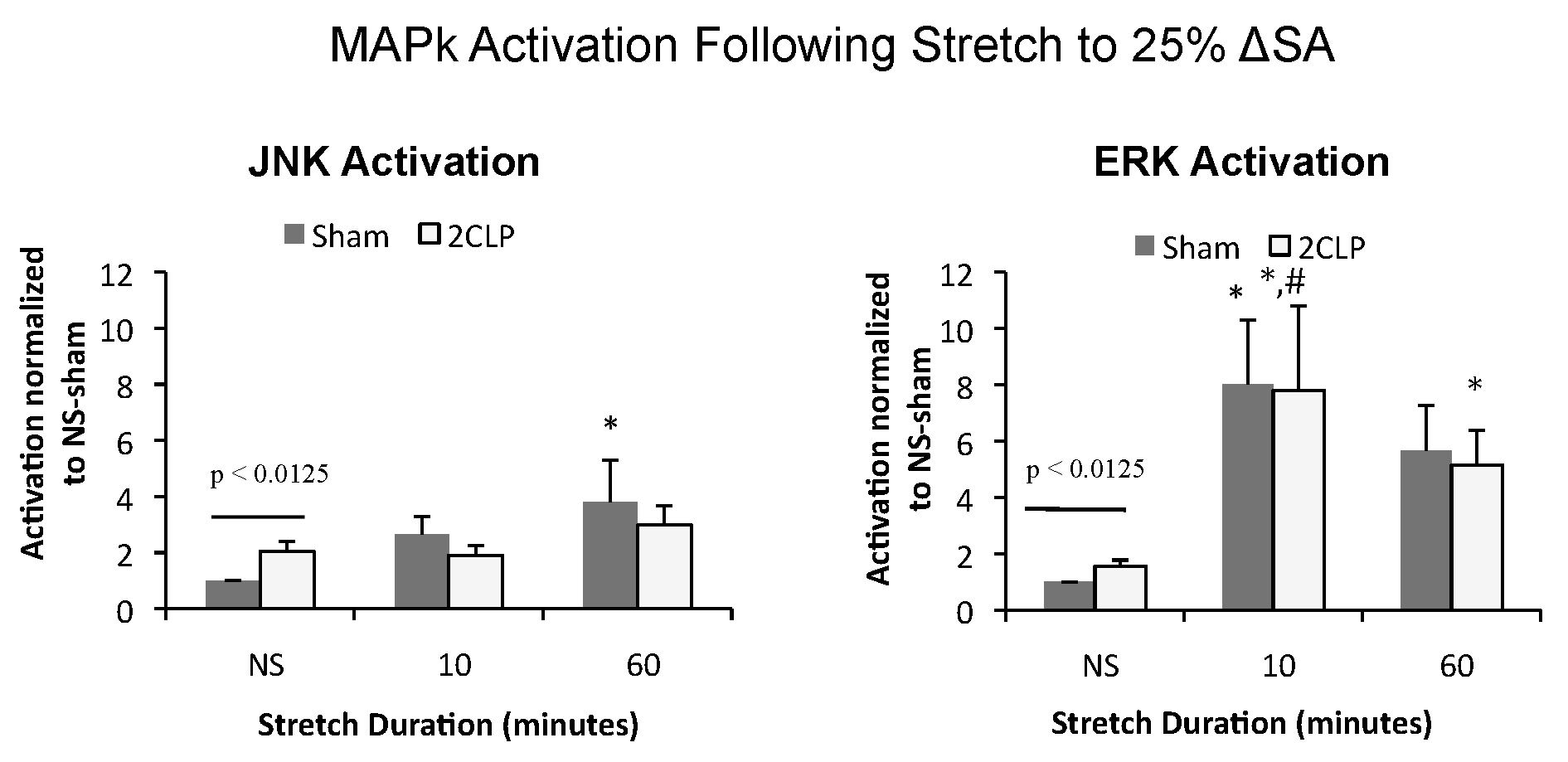

Supplement: Figure S1 — Stretch to 25% ΔSA induced significant phosphorylation of JNK in sham cells at 60 minutes compared to unstretched (NS) (*, p<0.05), but did not induce JNK phosphorylation at either time point. Stretch to 25% ΔSA for 10 minutes induced significant ERK phosphorylation in both sham and 2CLP monolayers compared to unstretched sham only (*, p<0.05), or to both unstretched sham and unstretched 2CLP (*,#, p<0.05). At 60 minutes of stretch, ERK phosphorylation had subsided in 2CLP monolayers, and was no longer significant from any other time point, while ERK phosphorylation in sham monolayers was still significantly elevated above unstretched sham. (mean ± SE, N≥6 isolations). (TIFF) [file pone.0038748.s001.tiff]

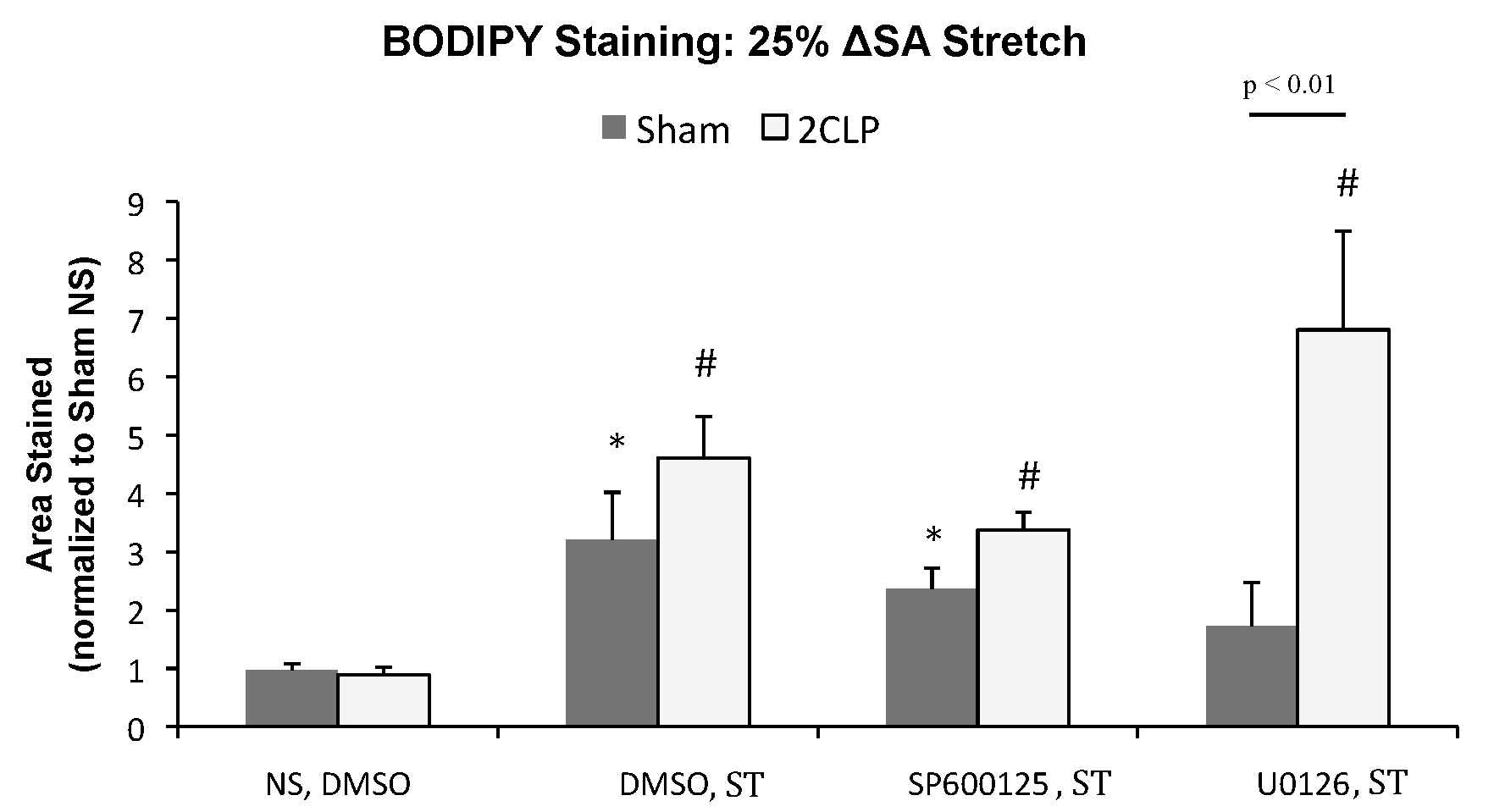

Supplement: Figure S2 — Inhibition of MAPk signaling (JNK-SP600125 20 µM, ERK-U0126 10 µM) did not prevent stretch-induced permeability increases in 2CLP monolayers when stretched (ST) to 25% ΔSA. ERK inhibition prevented significant permeability increases in sham monolayers only, when stretched to 25% ΔSA. JNK inhibition did not prevent significant permeability increases in sham and 2CLP monolayers stretched to 25% ΔSA. All stretched for 60 minutes. *significantly greater than sham unstretched (NS), #significantly greater than both sham and 2CLP NS, p<0.05 (mean ± SE, N≥2 isolations). (TIFF) [file pone.0038748.s002.tiff]

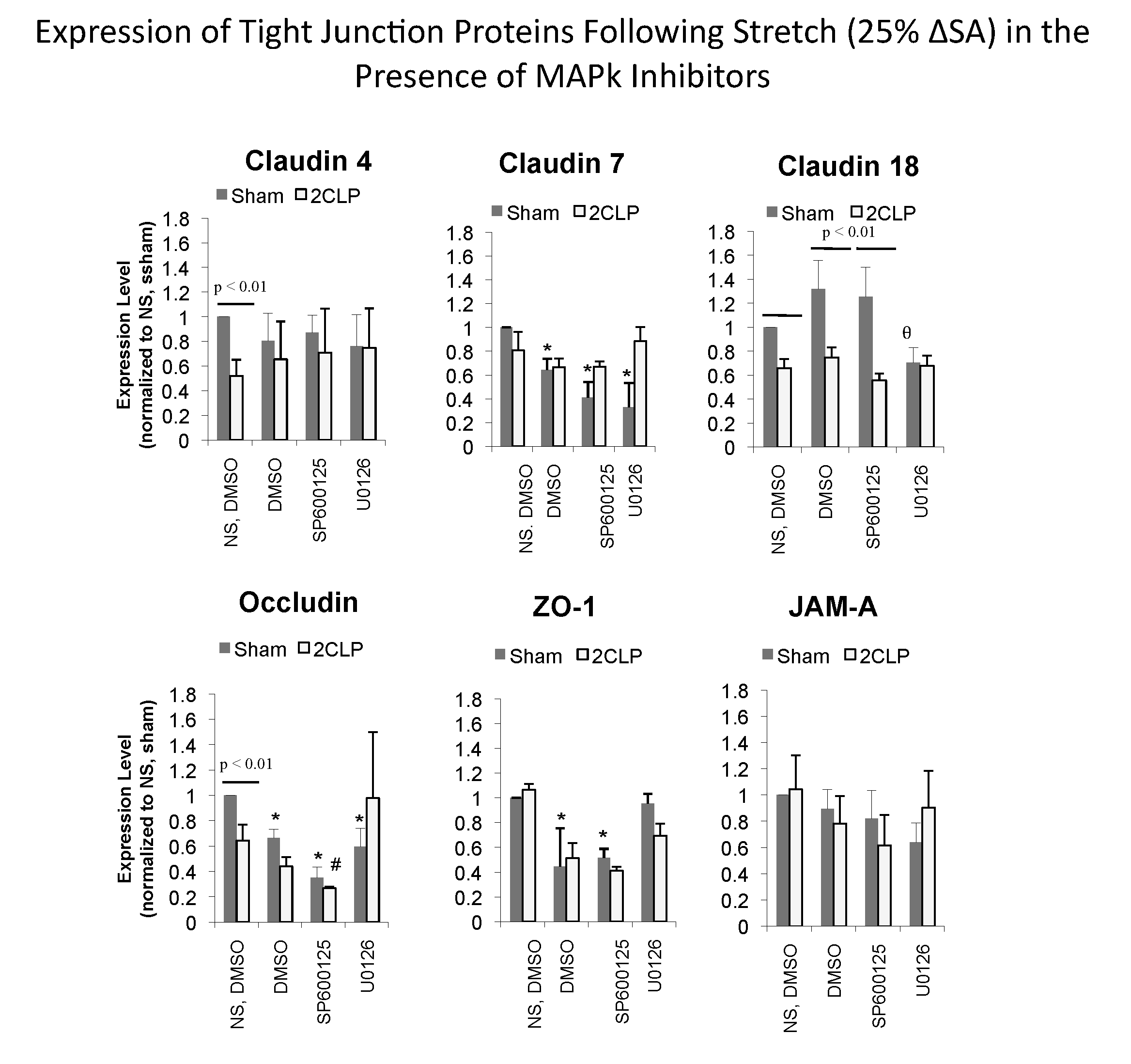

Supplement: Figure S3 — Inhibition of ERK activation in sham monolayers during stretch to 25% ΔSA for 60 minutes significantly reduces expression of claudin 18 compared to levels during stretch with DMSO treatment (θ). The stretch-induced reduction in sham treated with DMSO ZO-1 is reversed with ERK inhibition; however, this difference was not statistically significant. In 2CLP monolayers, inhibition of JNK activation during stretch significantly reduced occludin levels compared to unstretched (NS) 2CLP (#). *significantly different from DMSO NS, bar indicates significantly different from sham, p<0.05 (mean ± SE, N≥3 isolations). (TIFF) [file pone.0038748.s003.tiff]
